# An efficient and lightweight detection method for stranded elastic needle defects in complex industrial environments using VEE-YOLO

**DOI:** 10.1038/s41598-025-85721-9

**Published:** 2025-01-22

**Authors:** Qiaoqiao Xiong, Qipeng Chen, Saihong Tang, Yiting Li

**Affiliations:** 1 Department of Mechanical and Manufacturing Engineering, Faculty of Engineering, Universiti Putra Malaysia, Serdang, 43400 Selangor Malaysia; 2 Department of Mechanical and Electronic Engineering, Guizhou Communications Polytechnic University, Guiyang, 551400 China; 3 School of Mechanical Engineering, Guiyang University, Guiyang, 550005 China; 4 School of Mechanical Engineering, Guizhou University, Guiyang, 550025 China; 5 Key Laboratory of Advanced Manufacturing Technology, Ministry of Education, Guizhou University, Guiyang, 550025 China; 6 College of Big Data Statistics, Guizhou University of Finance and Economics, Guiyang, 550025 China

**Keywords:** Mechanical engineering, Computer science

## Abstract

Deep learning has achieved significant success in the field of defect detection; however, challenges remain in detecting small-sized, densely packed parts under complex working conditions, including occlusion and unstable lighting conditions. This paper introduces YOLOv8-n as the core network to propose VEE-YOLO, a robust and high-performance defect detection model. Firstly, GSConv was introduced to enhance feature extraction in depthwise separable convolution and establish the VOVGSCSP module, emphasizing feature reusability for more effective feature engineering. Secondly, improvements were made to the model’s feature extraction quality by encoding inter-channel information using efficient multi-Scale attention to consider channel importance. Precise integration of spatial structural and channel information further enhanced the model’s overall feature extraction capability. Finally, EIoU Loss replaced CIoU Loss to address bounding box aspect ratio variability and sample imbalance challenges, significantly improving overall detection task performance. The algorithm’s performance was evaluated using a dataset to detect stranded elastic needle defects. The experimental results indicate that the enhanced VEE-YOLO model’s size decreased from 6.096 M to 5.486 M, while the detection speed increased from 179FPS to 244FPS, achieving a mAP of 0.926. Remarkable advancements across multiple metrics make it well-suited for deploying deep detection models in complex industrial environments.

## Introduction

Deep learning object detection technology is a hot research direction in the fields of artificial intelligence and intelligent manufacturing^1–3^. Due to its strong feature learning capability, flexible application scenarios, and significant advantage of being less affected by factors such as shape, size, and texture, deep object detection technology has been applied to the defect detection task of production workpieces^4–6^. However, this kind of model is mostly used in the engineering scene with a single production mode and fixed detection object. The reason is mainly attributed to two points: First, in many precision manufacturing scenarios that require defect detection, the detection process is easily affected by factors such as small part sizes, reflective light sources, and high object densities. These challenges make it difficult to intelligently and accurately detect specific defects from such complex samples. Then, hardware deployment space is generally limited in manufacturing settings. In most cases, algorithms or models need to be integrated into embedded controllers, and there is a high demand for both accuracy and speed in the detection process. However, deploying more complex models on edge devices is challenging, and lightweight object detectors struggle to achieve higher accuracy. These issues hinder the development of deep object detection technology in the field of defect detection. The defect detection segment of stranded elastic needles is a good example to illustrate such issues. Stranded elastic needles are primary connector components of high-end electronic devices. They are made by twisting 10 strands of copper alloy wires, measuring 6–7 mm in length, with a diameter of less than 0.2 mm. These components find extensive application in significant projects such as human spaceflight missions, lunar exploration, BD satellite navigation systems, large aircraft, and high-resolution earth Observing Systems. However, the production process of stranded elastic needles includes wire winding, straightening, cutting, spot welding, punching, assembly crimping, assembly pressing, assembly straightening, assembly bulge adjustment, quality inspection, and other processes. Due to errors in the manufacturing process, various defects can arise in the products. It is of great practical significance to apply cutting-edge deep target technology to the actual production and scientific research of such precision parts to overcome several problems in the quality inspection process, such as small size, high density, and unfixed detection scenes.

This article focuses on the issues, aiming to enhance the generality and effectiveness of deep object detection models. The key contributions of this manuscript are summarized below:

1. Targeted reduction of parameter redundancy within the Neck section’s standard convolution, replacing standard convolutional structure with depthwise separable convolution (DSC)^7^ to boost network processing speed, and introducing GSConv^8^ to address the weaker feature extraction capability of DSC, ensuring a high-quality feature extraction process. Constructing the VOVGSCSP module feature fusion mechanism with GSConv as the reference unit, emphasizing feature reusability to create and strengthen more effective feature engineering.
2. The construction of an efficient multi-scale attention (EMA) mechanism^9^ encodes inter-channel information to consider the importance of different channels, integrating precise spatial structural information with channel information, thereby enhancing the overall feature extraction capability of the model.
3. Considering the issue of arbitrary aspect ratio changes of bounding boxes and the imbalance between difficult and easy samples, replacing the original network’s CIoU Loss^10^ with EIoU Loss^11^ enhances the overall performance of the detection task.

The remaining sections of this paper are organized as follows: The “Related work” section reviews previous relevant work. The “Methodology” section introduces an improved stranded elastic needle defect detection model (VEE-YOLO), providing a comprehensive explanation of the model’s structural components and operational mechanisms. In the “Experiment” section, the experimental environment and parameter settings are initially presented. Subsequently, model improvement experiments and comparative experiments are conducted on the stranded elastic needle defect detection dataset to comprehensively validate the feasibility of the proposed approach. In the “Discussion” section, the current research findings are summarized, and the results of this study are compared with existing literature, along with a discussion of the potential limitations of this research. The “Conclusion” section summarizes the work done in this paper and provides an outlook on future research directions.

## Related work

Influenced by factors such as production equipment, process flows, and manufacturing environments, defects in various processes are inevitable in production. Accurate and efficient detection of these defects holds significant research significance. Deep object detection methods can be divided into two-stage object detection algorithms, represented by Faster R-CNN^12^, and one-stage object detection algorithms, represented by the YOLO series^13–22^. The former requires training and generating candidate boxes that may contain objects, followed by refining the detection results. In contrast, the latter directly uses the features extracted in the backbone network to predict the target category and location. There has been a lot of research on this technique for defect tasks. For instance, Cha et al.^23^ proposed a structural visual detection method based on Faster R-CNN to realize the simultaneous detection of crack defects in concrete and steel structures. The study developed a self-built dataset, annotating five types of damaged concrete cracks, two levels of steel corrosion, and corroded bolt samples. To address the issue of poor real-time performance of Faster R-CNN for real-time target detection, this approach employed the structurally simple ZFNet as the backbone. The results indicate that the average precision (AP) for the five types of damage has surpassed 80%, with a mean average precision (mAP) of 87.8%. Hu et al.^24^ proposed a novel unsupervised method based on deep convolutional generative adversarial networks (DCGANs) for detecting fabric defects. This method decomposes image-level reconstruction into patch-level reconstruction, optimizing the reconstructed images using normalization and error maps. Subsequently, the residual maps between the reconstructed images and the original images are computed to determine the defect locations. Tao et al.^25^ addressed the critical issue of locating insulators and detecting defects only at specific scales or under specific lighting conditions. This study utilized drone-captured images of insulators and accurately identified insulator defects captured in real detection environments. By designing a novel deep convolutional neural network (CNN) cascade structure, they first detected insulators from natural scenes to eliminate background interference. Subsequently, defects were detected within the cropped insulator regions. He et al.^26^ proposed a deep learning defect detection system for steel plate defect detection, based on a multilevel-feature fusion network (MFN). This system employs convolutional neural networks to extract deep defect features from steel plate images and concatenates features from multiple levels, which can include more detailed defect location information. Experimental results demonstrate that fusing multiscale features can effectively improve the accuracy of steel plate defect detection.

Many of the methods require significant memory and computational resources, which need improvement and optimization for most industrial scenarios. Their universality needs enhancement. Therefore, scholars have already researched the lightweight of deep detection models to address these challenges. For instance, Deng et al.^27^ proposed an insulator defect detection method based on the one-stage detector YOLOv4. This method deploys a deep object detection network on edge devices, first achieving a lightweight improvement by replacing the original Darknet with MobileNetv3. Additionally, the particle swarm optimization (PSO) algorithm was introduced, enabling efficient partitioning of the deep neural network within limited time and computational resources. Liu et al.^28^ proposed a weld defect detection method based on X-ray images, called lighter and faster YOLO (LF-YOLO). This method incorporates a reinforced multiscale feature (RMF) module to facilitate the feature fusion process in deep convolutional networks. Additionally, an efficient feature extraction (EFE) module is introduced to handle input data with extremely low power consumption. These two methods complement each other, enhancing detection accuracy and speed, thereby improving the practicality of the network in real-world industrial applications. To accurately detect surface defects in precision devices such as surface-mounted device light-emitting diodes (SMD LEDs), Chen et al.^29^ designed a defect detection model based on the YOLOV3 detector. This model can identify defects such as missing components, incorrect placement, reverse polarity, and missing wires. By using densely connected convolutional networks (DenseNet) as the backbone, the model enhances feature engineering, thereby improving detection accuracy while maintaining real-time performance. Yang et al.^30^ proposed a real-time object recognition method for 0.8 cm darning needles and KR22 bearings in complex industrial settings. This method is based on an image data augmentation algorithm with directional flipping and a precision component classification algorithm with non-maximum suppression, culminating in an improved YOLO V3 network. Experimental results demonstrate that the proposed method outperforms the YOLO V3 algorithm in terms of recognition accuracy and robustness. Yang et al.^31^ considered the significant impact of the properties of tiny parts and the environmental parameters of the defect detection system on its stability. They established a correlation model between the detection capability coefficient of the part system and the moving speed of the conveyor. Additionally, they proposed a defect detection algorithm for tiny parts based on the single shot detector (SSD) and deep learning. By integrating an industrial real-time detection platform with a mechanical component defect detection algorithm based on intermediate variables, they addressed the issue of missed defect detections.

## Methodology

According to network width and network depth, YOLOV8 is divided into four detection models: YOLOV8-l, YOLOV8-m, YOLOV8-s, and YOLOV8-n. Considering the possible limitations of hardware when using deep learning networks in industrial scenarios, and the universal applicability of network deployment in diversified scenarios, this article selects YOLOV8-n, which has the smallest number of parameters and the fastest detection speed, as the baseline network.

### YOLOV8-n baseline network

**Fig. 1:**
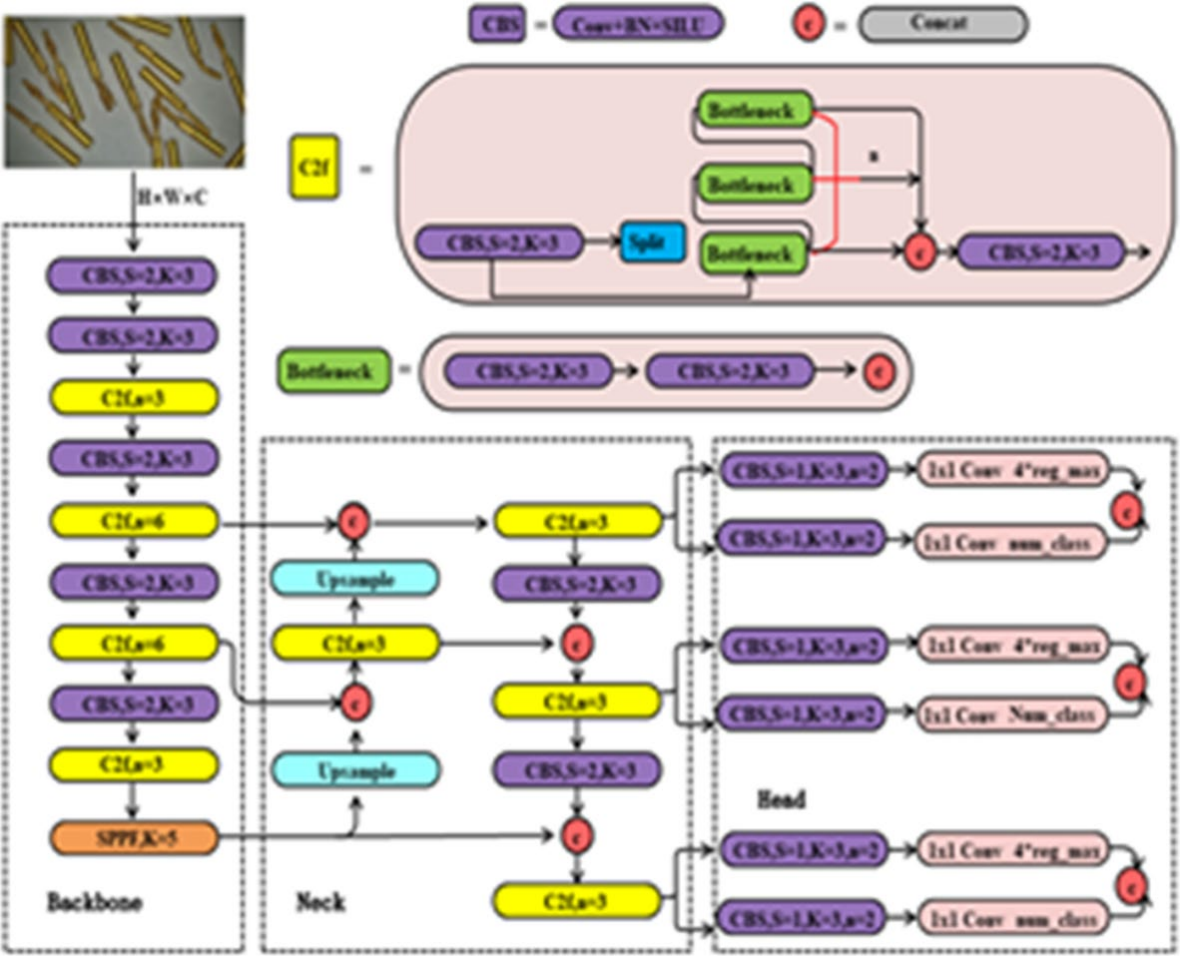
YOLOv8 basic structure.

As shown in Fig. 1, YOLOV8-n continues the classic structure of the YOLO series. This structure employs operations like deep residual convolutions and normalization from the backbone to continuously extract effective features and reduce the image dimensions. Subsequently, the features are sent to the neck, where a path aggregation network-feature pyramid networks (PAN-FPN)^32^ structure is used from the perspective of feature fusion to perform multi-scale feature engineering, resulting in features of three different sizes: large, medium, and small. Finally, the fused features are fed into the detection head, where a Decoupled Head is employed to accomplish high-precision classification and bounding box regression tasks, ultimately producing the detection results.

In Fig. 1, the cross stage partial network fusion (C2f) module within the backbone is an improvement over the original C3 module, drawing inspiration from the ELAN structure in YOLOv7, which possesses advantages in richer gradient information. This module achieves a balance between lightweight design and enhanced gradient flow information by reducing standard convolutional layers and making full use of the Bottle-neck module’s gradient expansion branch.

The PAN-FPN module in Neck consists of two parts: feature pyramid networks (FPN) and path aggregation network (PAN)^33^. In this context, the former is responsible for transmitting the rich semantic features of the top layer to the bottom layer, and the latter is responsible for transmitting the precise positioning information of the bottom layer to the top layer. While FPN and PAN complement each other, they also fuse features of the same size between them to improve the accuracy of predicted bounding box positions and the precision of predicted bounding box categories in the model.

### Improvements to the neck part

**Fig. 2:**
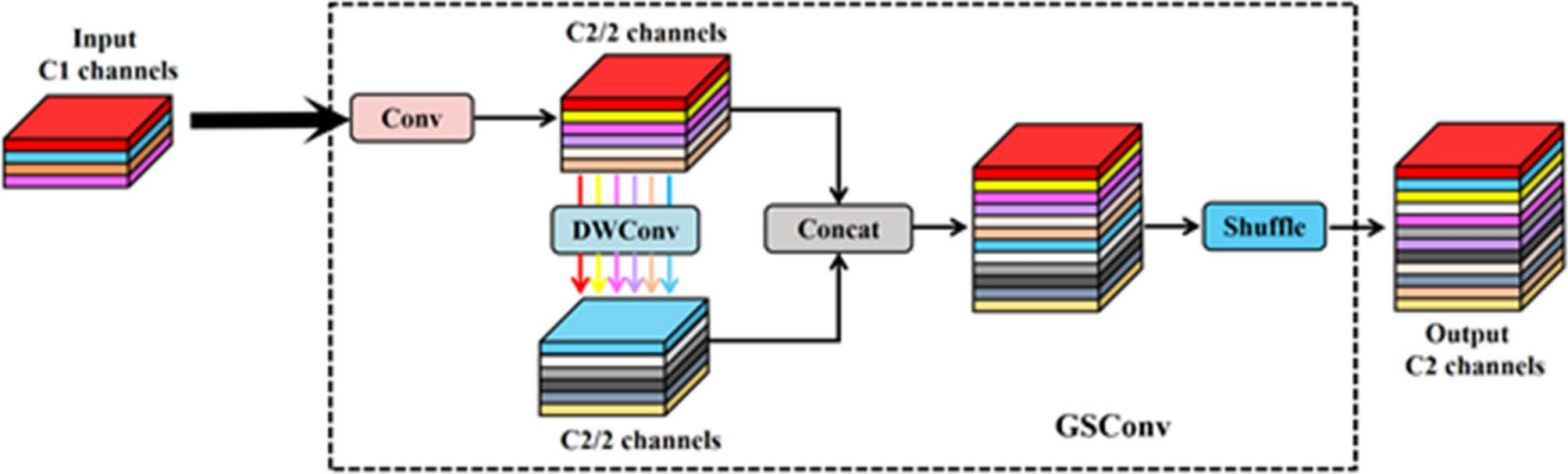
GSConv basic structure.

DSC can split all input features into single-channel feature maps and stack them back together after performing single-channel convolution on each feature map separately. Compared with the standard convolution, the operation process of DSC reduces many parameters and realizes the separation of channels and regions, which improves the robustness of the convolution process to some extent. However, since DSC is completely performed in a two-dimensional plane, and this operation mainly performs convolution operations independently for each channel of the input layer, this process does not effectively utilize the feature information of different channels at the same spatial position, resulting in its feature extraction ability being weak. Based on the above problems, this paper improves the overall performance of the Neck part from the following two aspects:

On one hand, the GSConv concept is introduced. The core idea of this structure is to combine standard convolution, DSC, and shuffle mechanisms so that this new convolution structure can approximate the effect of standard convolution as much as possible while retaining the parameter advantages of DSC. In Fig. 2, GSConv converts the feature information generated by standard convolution into each part of the information generated by DSC by using the shuffle mechanism. The process allows the information from standard convolution operations to mix with that of DSC and exchange local feature information across different channels, addressing the significant limitation of DSC mentioned earlier, where it didn’t effectively utilize feature information from different channels at the same spatial location.

**Fig. 3:**
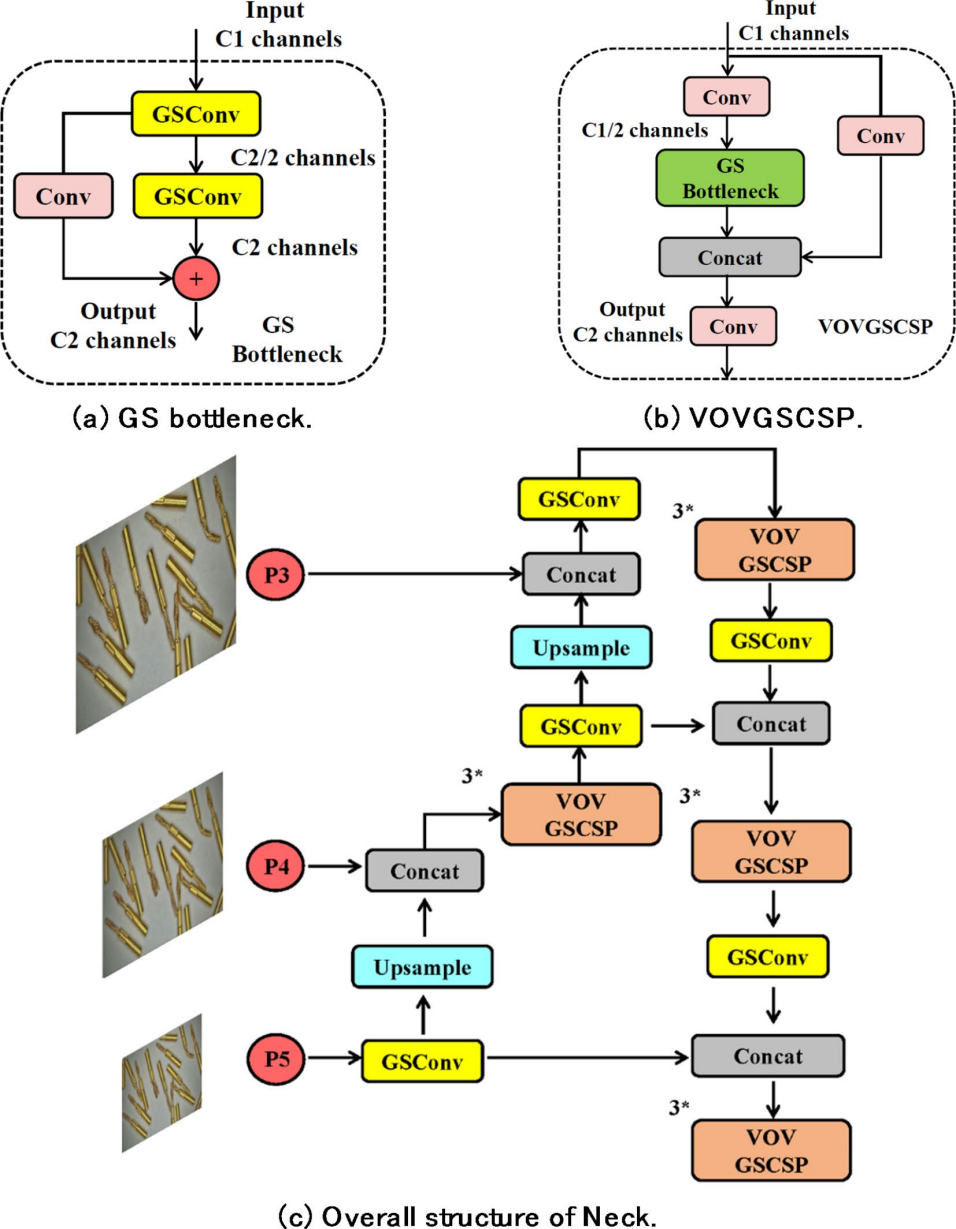
The improvement plan at the Neck.

On the other hand, this structure is used as a baseline unit to design the GS bottleneck (shown in Fig. 3a). This structure takes input features through two layers of GSConv with progressively increasing convolutional kernel counts, resulting in feature maps of the standard output size. These feature maps form a residual structure with feature maps obtained through standard convolution, enhancing the feature extraction capability of the entire module. In addition, the design of the VOVGSCSP structure shown in Fig. 3b, further integrates the features of GS bottleneck and standard convolution, replaces the C2f structure in the original YOLOv8-n Neck with this structure, and strengthens the features while reducing the number of detection network parameters Engineering to improve detection accuracy. The entire Neck section is depicted in Fig. 3c.

### Improvement in feature extraction method

In addition to the conventional Conv2D + BatchNorm + SiLu (CBS) convolutional structure, YOLOv8 utilizes a significant amount of C2f structures to enhance gradient flow information, extracting more comprehensive hierarchical image features. However, convolutional structures often have the following limitations: First, while CNNs can enhance learned feature representations by extending the depth of convolutional layers, continually expanding the number of convolutional layers in a model consumes significant computational resources and memory. This trade-off is often not worthwhile in model performance evaluation. Second, the core idea of convolutional structures is to capture local features within the receptive field, inherently limiting the representation of features across multiple dimensions.

In recent years, attention mechanisms have garnered widespread attention in the field of object detection. Due to their flexible structural characteristics and diverse feature representation dimensions, attention mechanisms not only make features more discriminative but also comprehensively showcase features from aspects such as convolutional channels and spatial dimensions, breaking the limitations of convolutional structures. Attention mechanisms can be easily integrated into the backbone architecture of neural networks, and when combined with convolutional neural networks, they exhibit strong advantages and robustness in various multi-class tasks. From recent advancements in attention mechanisms, it is evident that cross-dimensional interactions contribute to channel or spatial attention prediction. Hence, this paper draws inspiration from the EMA, emphasizing enriching the feature extraction of YOLOv8 from the perspective of leveraging the strengths of attention mechanisms. This integration aims to complement convolutional operations with efficient attention, synergistically improving the quality of feature engineering for defect detection tasks.

The implementation process of EMA is as follows: Firstly, given the input feature $\:X\in\:{R}^{C\times\:H\times\:W}$, it is divided into $\:G$ parts along the channel dimension to enhance the learning process for different semantic representations. Therefore, $\:X$ can be represented as $\:X=[{X}_{0},{X}_{2},\dots\:\dots\:{X}_{G-1}],{X}_{i}\in\:{R}^{C//G\times\:H\times\:W}$.

Next, by designing two attention branches to aggregate multiscale spatial structural information. Branch 1 refers to the coordinate attention (CA) mechanism, utilizing two 1D global average pooling operations to encode features in the two spatial directions and concatenate these encoded features along the image’s height direction. Subsequently, the vectors obtained through convolutional outputs undergo a Sigmoid function to attain their nonlinear mapping. The two-channel attention mappings are then aggregated through simple multiplication, facilitating cross-channel feature interaction effects. Branch 2, on the other hand, employs straightforward 3 × 3 convolutional operations in conjunction with Branch 1, effectively establishing short-term and long-term dependencies between information.

Finally, the outputs of the two branches are introduced. They employ 2D global average pooling and the nonlinear function softmax to respectively encode and fit linear transformations for the global spatial information within the two tensors. By performing matrix multiplication on the concurrently processed outputs of these two branches and adding the results together after activation, directly applying them to the original feature map, the attention mechanism output features are obtained. The formula for 2D global average pooling is as follows:

1 $$z = \frac{1}{{H \times W}}\sum\limits_{j}^{H} {\sum\limits_{i}^{W} {x(i,j)} }$$

**Fig. 4:**
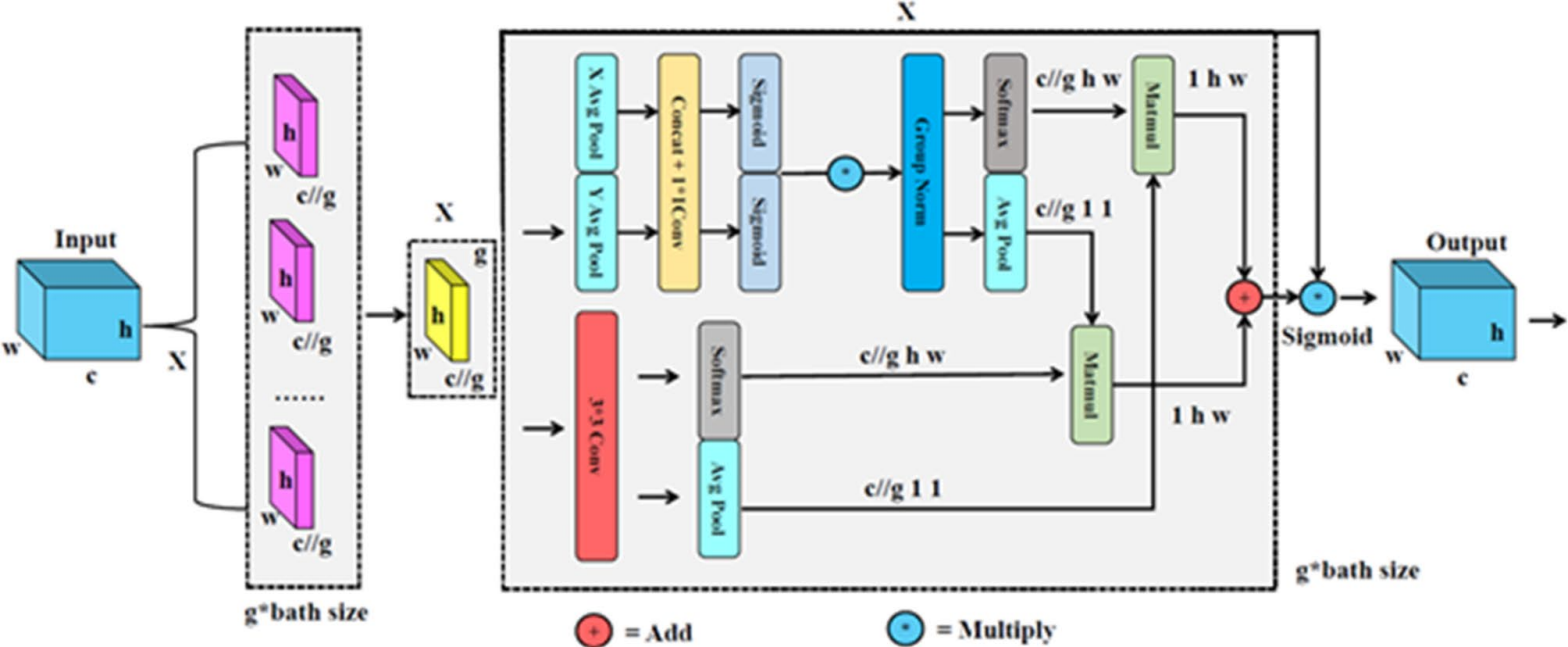
The implementation process of EMA.

EMA not only encodes information among channels to consider their respective importance but also combines precise spatial structural information with channel information, enhancing the overall feature extraction capability of the model. Its operational process is illustrated in Fig. 4.

### Improvement of the loss function

In YOLOv8, the anchor-free concept is utilized, leading to significant changes in its loss function compared to the traditional YOLO framework. The components of its loss function include the classification and regression parts, both of which interact to jointly optimize the network weights. Specifically, the classification loss and regression loss use binary cross-entropy loss (BCEL), distribution focal loss (DFL)^34^, and bounding box regression loss (BBRL)^35^. The loss function for YOLOv8 can be represented as:

2 $$\:{f}_{loss}={\lambda\:}_{1}{f}_{BCEL}+{\lambda\:}_{2}{f}_{DFL}+{\lambda\:}_{3}{f}_{BBRL}$$

Among them, the predicted category loss is essentially cross-entropy loss, expressed as:

3 $$f_{{BCEL}} = weight\left[ {class} \right]\left( { - x[class] + log\left( {\sum\limits_{j} {exp(x[j])} } \right)} \right.$$

In formula (3): “$\:class$” represents the number of classes; “$\:weight\left[c\right]$” denotes the weight for each class; the input “$\:x$ " is the probability value that has undergone sigmoid activation.

DFL is an optimization of the Focal Loss, which generalizes the discrete classification results into continuous results through integration. The expression is as follows:

4 $$\:{f}_{DFL}\left({S}_{i},{S}_{i+1}\right)=-\left(\right({y}_{i+1}-y\left){log}({S}_{i}\right)+(y-{y}_{i}){log}({S}_{i+1}\left)\right)$$

In formula (4): “$\:{y}_{i}$” and “$\:{y}_{i+1}$” represent values close to the continuous label “$\:y$” on the left and right sides, satisfying $\:{y}_{i}<y<{y}_{i+1}$, $\:y={\sum\:}_{i=0}^{n}P\left({y}_{i}\right){y}_{i}$; where “$\:P$ " can be achieved through a softmax layer containing “$\:n+1$”, and “$\:P\left({y}_{i}\right)$ " is equivalent to “$\:{S}_{i}$”.

The original YOLOv8 used CIoU Loss as the Bounding Box Regression Loss. However, CIoU mainly reflects the differences in the height and width of the predicted box compared to the ground truth, but it does not indicate the true differences in height and width with their respective confidences. Therefore, this sometimes hinders the effective optimization of the model’s similarity. The specific reasons are as follows: According to the definition of the aspect ratio $\:\upsilon\:$, it is found that when the ratio of the width and height of the predicted frame to the real frame satisfies $\:\left\{\right(w=k{w}_{gt},h=k{h}^{gt})\left|k\right.\in\:{R}^{+}\}$, the relative proportion penalty item added by CIoU will not come into effect. Additionally, as shown in the formulas (5) and (6) for the width and height of the predicted box relative to $\:\upsilon\:$:

5 $$\frac{{\partial \upsilon }}{{\partial w}} = \frac{8}{{\pi ^{2} }}\left( {arctan\frac{{w^{{gt}} }}{{h^{{gt}} }} - arctan\frac{w}{h}} \right) \times \frac{h}{{w^{2} + h^{2} }}$$

6 $$\frac{{\partial \upsilon }}{{\partial h}} = - \frac{8}{{\pi ^{2} }}\left( {arctan\frac{{w^{{gt}} }}{{h^{{gt}} }} - arctan\frac{w}{h}} \right) \times \frac{h}{{w^{2} + h^{2} }}$$

From this, you can derive $\:\frac{\partial\:\upsilon\:}{\partial\:w}=-\frac{h}{w}*\frac{\partial\:\upsilon\:}{\partial\:h}$, indicating that the gradient values $\:\frac{\partial\:\upsilon\:}{\partial\:w}$ and $\:\frac{\partial\:\upsilon\:}{\partial\:h}$ of the predicted box $\:w$ and $\:h$ have opposite signs. The opposite sign during training causes one of the values, either $\:w$ or $\:h$, to increase when the other must decrease during the box regression process. This constraint prevents both $\:w$ and $\:h$ from increasing or decreasing together during the box regression process, which hinders the model’s optimization. Therefore, the loss function must not only consider the aspect ratio of the bounding box but also address the issue of imbalanced hard samples.

EIoU effectively addresses the issue. Based on the penalty item of CIoU, the loss function separates the influence factors of the aspect ratios of the predicted and true bounding boxes and calculates the length and width of both separately. These speeds up convergence and enhances regression accuracy. Furthermore, due to the introduction of Focal Loss, EIoU addresses the issue of imbalance in bounding box regression tasks. It reduces the impact of anchor boxes with low overlap with the target bounding box, allowing the regression process to focus more on high-quality anchor boxes. The expression for EIoU is as follows:

7 $$\:{L}_{EIoU}={L}_{IoU}+{L}_{dis}+{L}_{asp}=1-IoU+\frac{{\rho\:}^{2}(b,{b}^{gt})}{({w}^{c}{)}^{2}+({h}^{c}{)}^{2}}+\frac{{\rho\:}^{2}(w,{w}^{gt})}{({w}^{c}{)}^{2}}+\frac{{\rho\:}^{2}(h,{h}^{gt})}{({h}^{c}{)}^{2}}$$

**Fig. 5:**
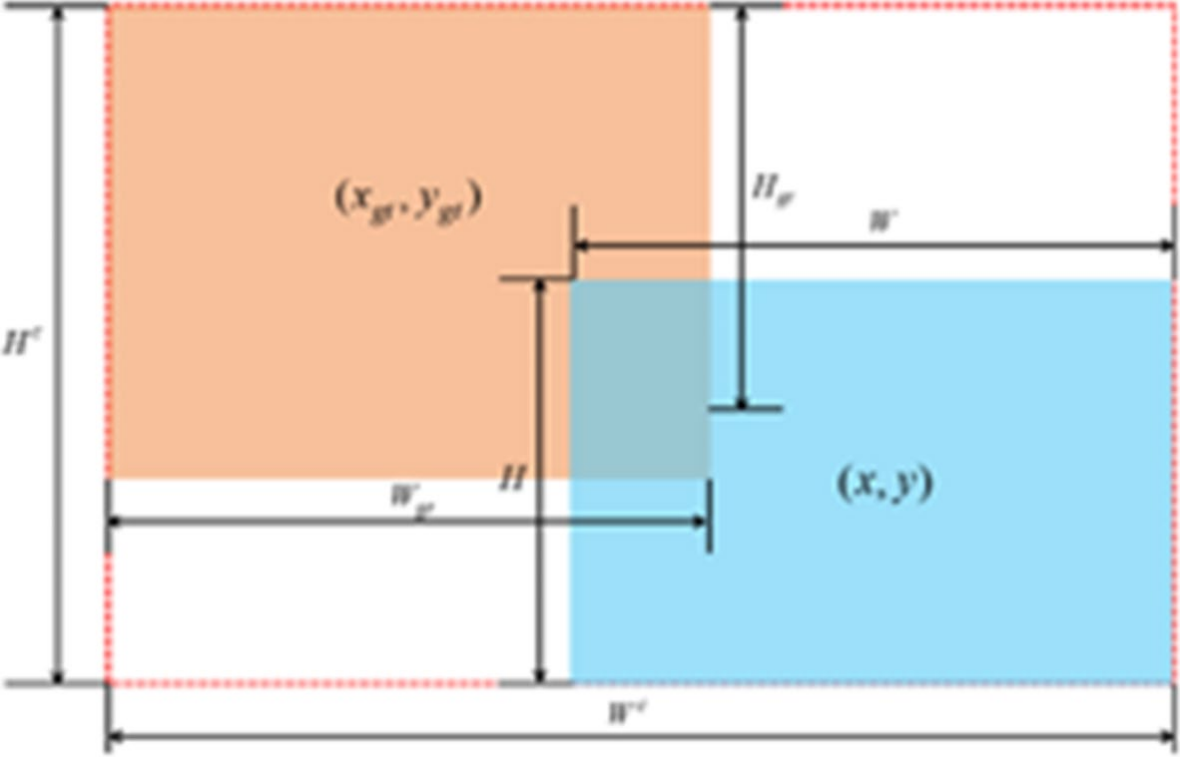
EIoU optimization effect illustration.

In formula (7), $\:{C}_{w}$ and $\:{C}_{h}$ represent the width and height of the minimum bounding box covering the predicted frame and the real frame. From the EIoU Loss formula, it can be seen that EIoU Loss includes three parts: the overlapping loss $\:{L}_{IoU}$ of the predicted frame and the real frame, The center distance loss between the predicted box and the real box is $\:{L}_{dis}$, and the width and height loss between the predicted box and the real box is $\:{L}_{asp}$. The first two parts of EIoU Loss continue the method in CIoU Loss, but the width and height loss directly minimize the difference between the width and height of the predicted frame and the real frame, making the convergence faster and having better positioning results. The optimization effect of EIoU is shown in Fig. 5.

## Experiment

### Dataset and preprocessing

**Fig. 6:**
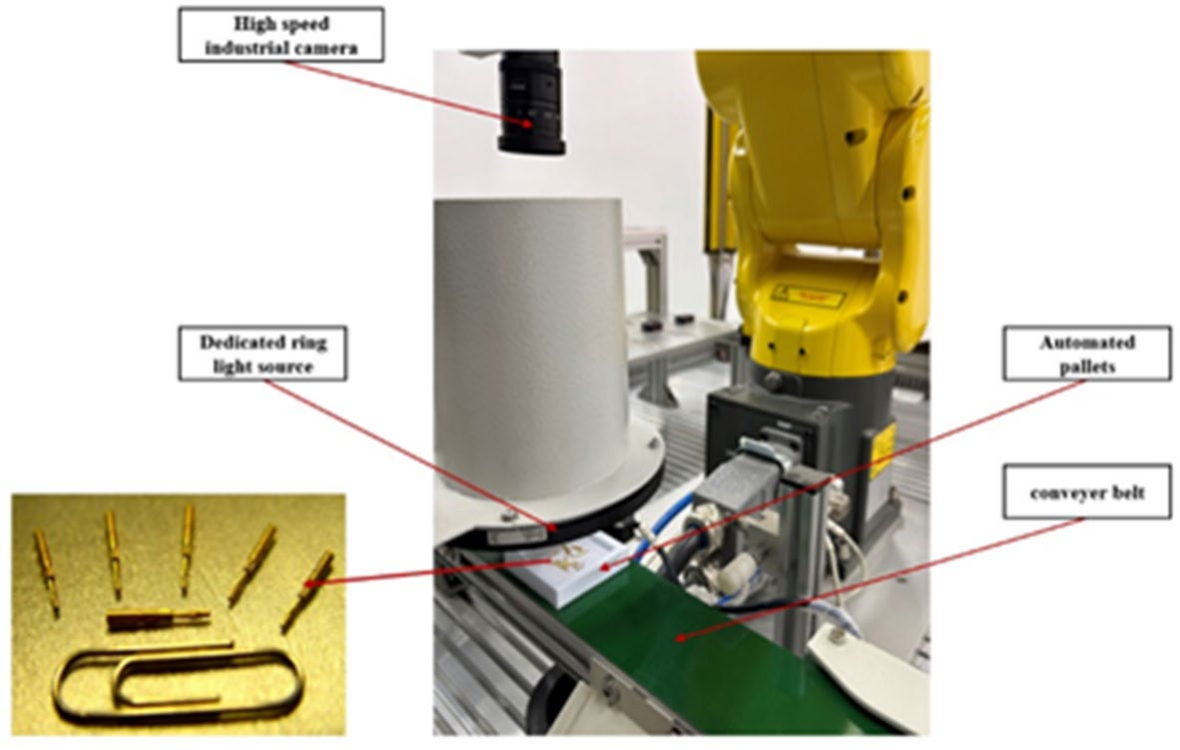
On-line defect detection device for stranded elastic needles.

Stranded elastic needles are a crucial connector component for high-end electronic devices, and their manufacturing process is highly intricate. A stranded elastic needle is composed of 10 strands of twisted copper alloy wires, with a length of approximately 6–7 mm and a diameter not exceeding 0.2 mm. Different from conventional inspection datasets, defect detection in stranded elastic needles can be challenging due to factors such as their small needle size, susceptibility to light reflection, and high density, all of which can affect the effectiveness of the inspection. Based on real-world conditions, a dataset for stranded elastic needle defect detection was created. To prevent factors like shooting angles, lighting, and backgrounds from affecting the detection performance, this dataset kept these three variables fixed and employed various imaging devices for capturing images in multiple scenarios and tasks. Specifically, includes variations in density (sparse and crowded), different types of defects (a mixture of 4 different defect types), and the capturing process using different imaging equipment (detailed in Fig. 6).

**Fig. 7:**
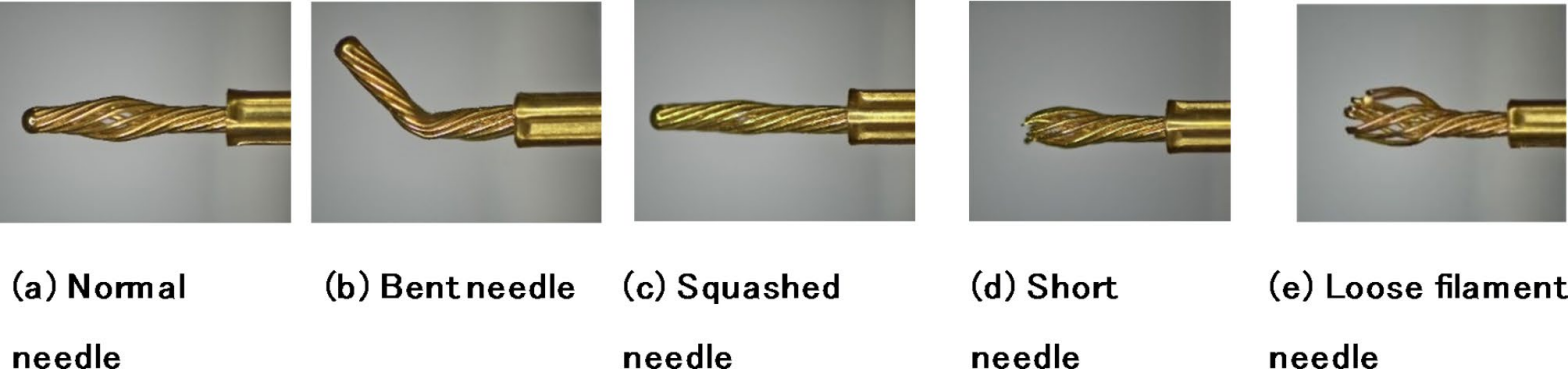
Stranded elastic needles defect dataset sample examples.

**Fig. 8:**
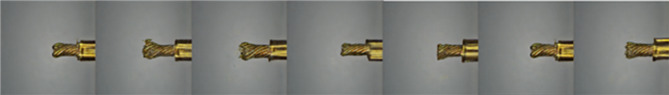
Some sample examples obtained by the improved DCGAN.

The stranded elastic needles defect dataset comprises images of five types: Normal needle、Short needle、Loose filament needle、Bent needle and Squashed needle (as shown in Fig. 7). Due to the insufficient number of images to support the robustness of deep learning, coupled with the significant characteristics of Stranded elastic needle defect detection, which generally exhibits minor differences in imaging effects and relatively fixed imaging forms, DCGAN^36^ is an unsupervised learning model designed to optimize the structure of generative adversarial networks (GAN)^37^ by incorporating deep convolutional neural networks. This aims to improve the quality of generated samples and accelerate convergence, resulting in more diverse and complex images. These images aid in training more robust detection models. Due to its excellent generation effects and the effective reduction of issues like gradient vanishing, DCGAN is widely used in the field of image data augmentation^23,38,39^. However, DCGAN may encounter problems such as detail loss, mode collapse, unstable generation quality, and training instability. These issues can be effectively mitigated by increasing the number of network layers, setting reasonable training parameters, and improving the loss function. Based on this, this paper employs DCGAN to augment the dataset, increasing the sample count from the original 792 images to 1500 images. To accurately evaluate the model’s performance, the augmented data is used solely for training the model. Some samples generated by DCGAN are shown in Fig. 8. Due to the deep convolutional neural networks’ thorough extraction of numerous features from the original dataset, the images generated by DCGAN are generally consistent with the types of sample images captured in the field.

The stranded elastic needles defect-enhanced dataset generated through improved DCGAN consists of a total of 1,500 images, with each defect category comprising 300 images. This dataset has been divided while considering class balance, thereby avoiding class imbalance issues and ensuring a fair and effective comparison between different detection models. The dataset has been split into a training set (1200 samples) and a test set (300 samples). Considering that each sample image contains multiple objects of various sizes and has a certain level of density, the authors have normalized the sample size to 608*608 pixels. This size setting offers several advantages: it ensures the imaging quality of the original image, laying the foundation for the feature extraction process without significantly sacrificing useful information. Additionally, it considers the real-time requirements of model detection, enabling the model to be deployed on edge devices for practical implementation. The experimental hardware and deep learning framework are as follows: an Intel(R) Core (TM) i9-12900 K processor with 16 cores and 24 threads, operating at a clock frequency of 3.19 GHz, and 32GB of RAM; a graphics processor is the Ge-Force RTX 3090Ti with 24GB of VRAM; the deep learning model framework is PyTorch 1.9.1 and Torchvision 0.10.1; the baseline version of YOLOv8 is Ultralytics 8.0.25. The model improvements based on YOLOv8 are built upon YOLOv8-n. This series of models scales down the original model in terms of network width and depth, resulting in models with fewer parameters, lower memory usage, and shorter inference times. They are well-suited for deployment on edge devices. To ensure fairness in the experiments since model improvements are involved, the training processes for all models maintain parameter consistency and do not utilize pre-trained weights. The critical parameter settings for the training process are outlined in Table 1.

**Table 1:** Training parameter setting table.

Parameters	Setup
Epochs	100
Batch size	16
Optimizer	SGD
NMS IoU	0.7
Initial learning rate	1e − 2
Final learning rate	1e − 4
Momentum	0.937
Weight-decay	5*1e − 4
Image scale	0.5
Image flip left-right	0.5
Mosaic	1.0
Image translation	0.1
Close mosaic	Last 10 epochs

## Model improvement experiment

### Subcategory performance

In this paper, improvements to YOLOV8-n are made for the defect detection process of stranded elastic needles as follows: Firstly, the model’s Neck section is enhanced. GSConv is introduced to address the weakness in feature extraction ability associated with DSC for defects. The VOVGSCSP module is constructed based on GSConv as the base unit to facilitate feature fusion, considering feature reusability, and enhancing more effective feature engineering. Secondly, improving the quality of feature extraction in the model. Encoding information among channels to consider the importance of different channels, and integrating precise spatial structural information with channel information, enhances the overall feature extraction capability of the model. Thirdly, improve the loss function of the model. Specifically, EIoU is used to replace CIoU as the Bounding Box Regression Loss, considering the free variation of aspect ratios of bounding boxes and the imbalance of difficult and easy samples. The two parts complement each other and jointly improve the detection accuracy and detection speed of the model. To scientifically demonstrate the above processes, here we will sequentially present the performance of YOLOv8-n, YOLOv8n + VOVGSCSP, YOLOv8n + VOV-GSCSP + EMA, and VEE-YOLO (YOLOv8n + VOVGSCSP + EMA + EIoU Loss) models in terms of various dimensions commonly used in the field of object detection: precision rate (P), recall rate (R), AP, mAP, frames per second (FPS), parameter count, and model size. The expressions and explanations for some of these metrics are as follows:

8 $$\:p=\frac{TP}{TP+FP}$$

9 $$\:R=\frac{TP}{TP+FN}$$

10 $$AP = \int\limits_{0}^{1} {P\left( R \right)dR}$$

11 $$mAP = \frac{1}{{classes}}\sum\limits_{1}^{{classes}} {\int\limits_{0}^{1} {P\left( R \right)dR} }$$

**Fig. 9:**
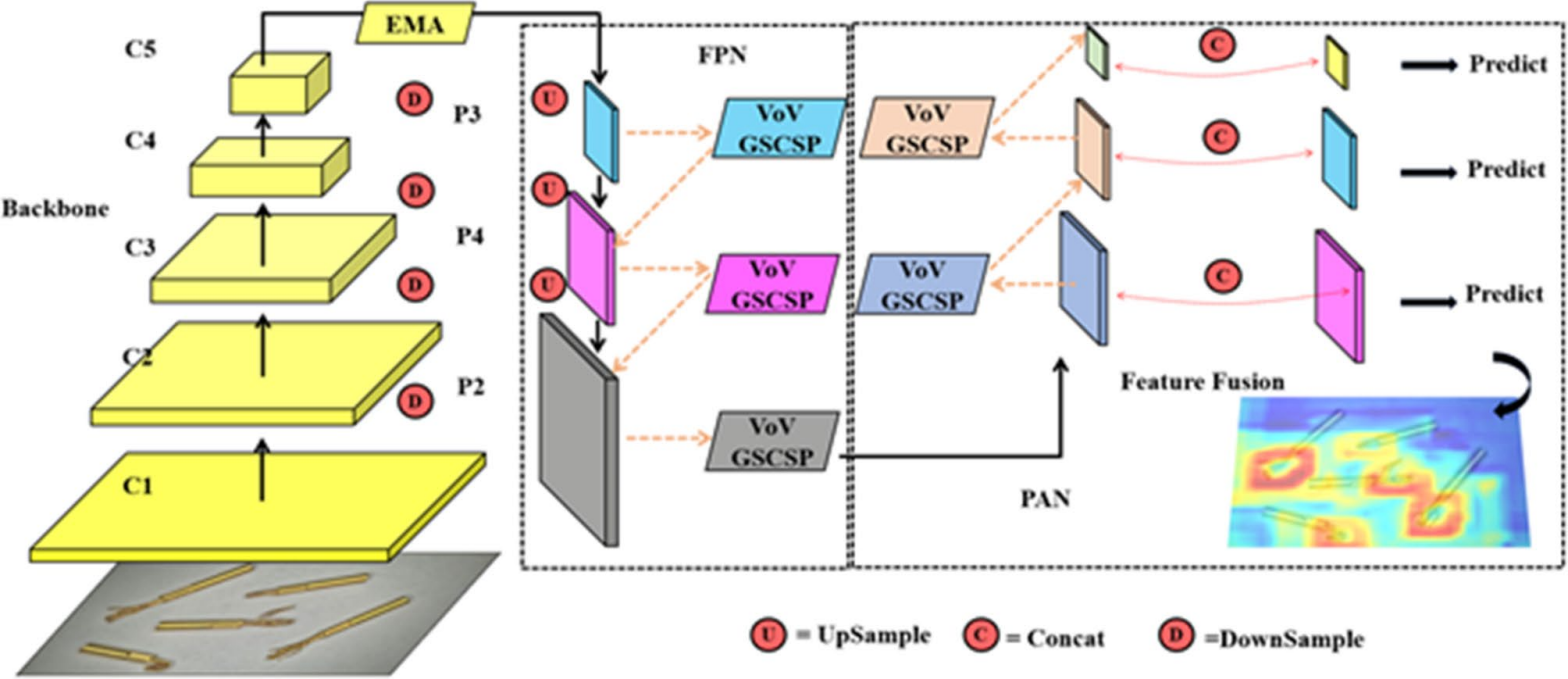
Improved VEE-YOLO network overall architecture.

**Fig. 10:**
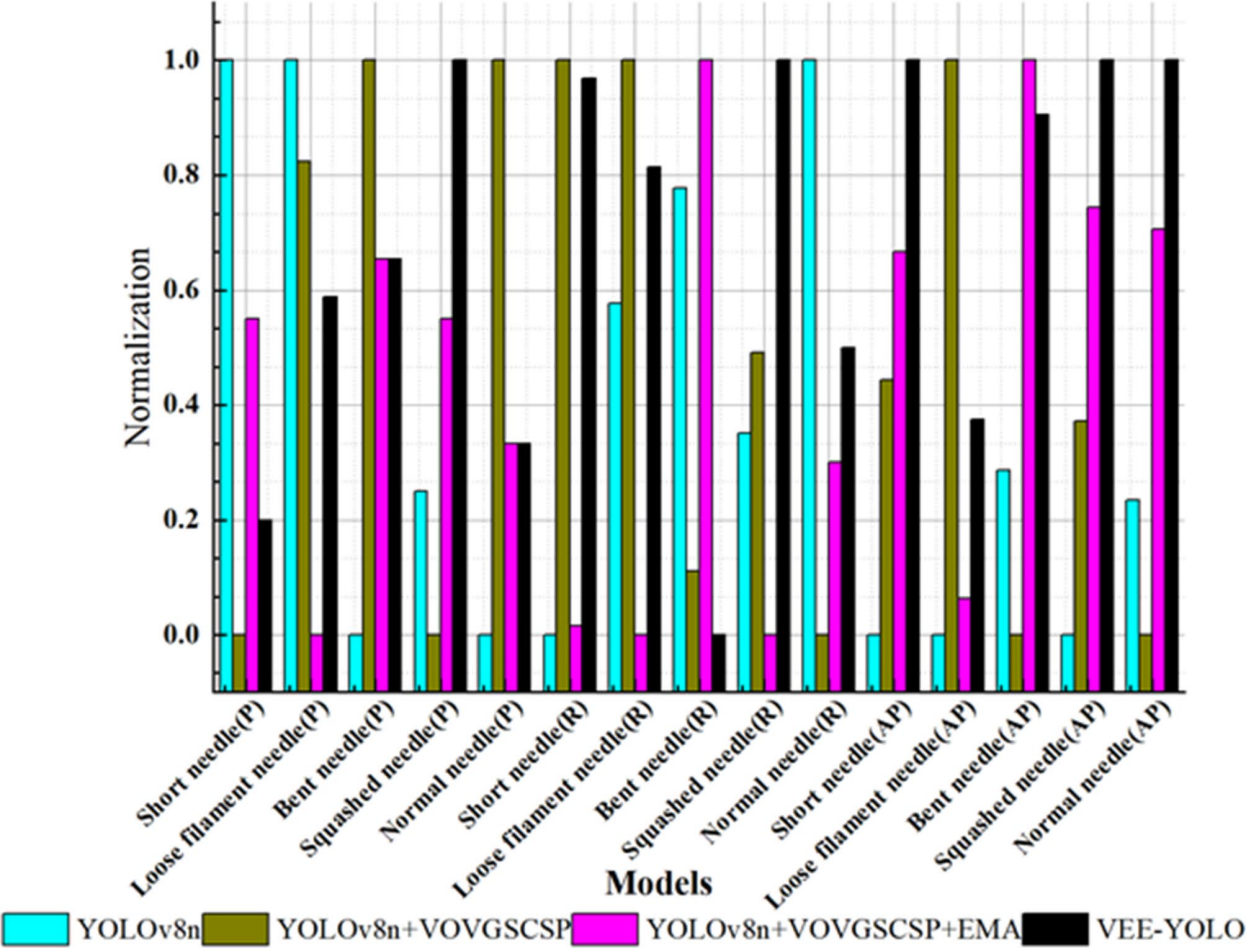
Segment category indicator lift effect.

**Fig. 11:**
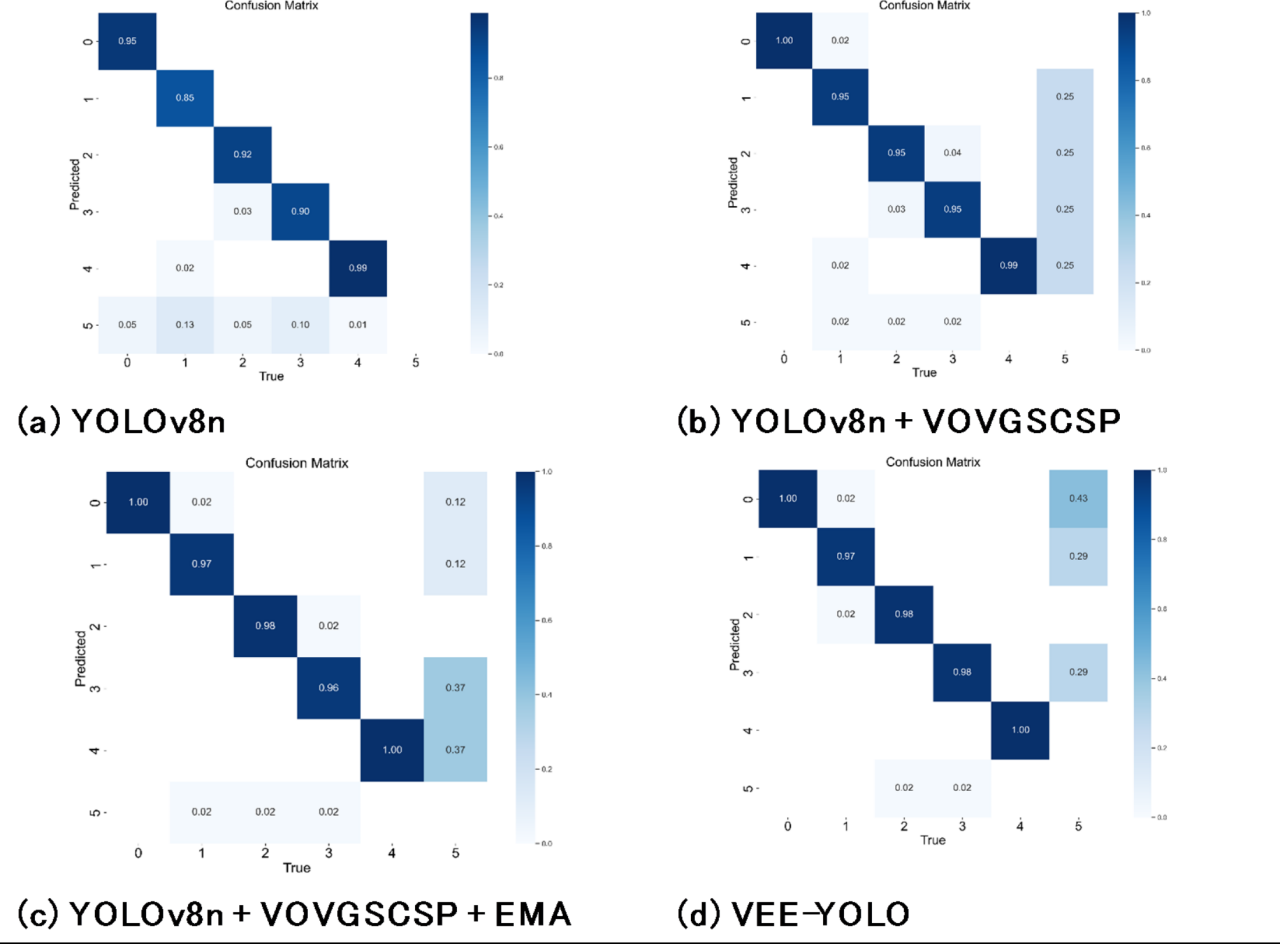
Confusion matrix diagrams for the four models.

In the formula: TP stands for true positives, which are correctly classified as positive instances. FP means that the wrong ones are classified into the positive category, including missed detection and detecting the background class as a defective class, that is, false positive cases; FN means that the wrong ones are classified into the negative class, that is, the case where the defect class is detected as a background class, that is, a false negative example; ‘classes’ refers to the number of categories or classes present in the dataset. The overall architecture of the improved VEE-YOLO network is shown in Fig. 9. The detailed classification results of the model on the test set are shown in Figs. 10 and 11.

0–5 in Fig. 11 respectively represent the five categories of data and the background category. Summarizing the segmentation indicators, that is, the classification results, shown in Figs. 10 and 11, the following conclusions can be drawn:

Firstly, as a baseline model, YOLOv8-n already demonstrates excellent performance. It achieves an AP of over 0.85 for each category and exhibits the best P values in certain categories such as Short Needle and Loose Filament Needle. Overall, YOLOv8-n shows precise intelligent detection performance on this dataset. Enhancing the detection speed of the model while maintaining its accuracy would undoubtedly increase the practicality of the algorithm.

Secondly, by incrementally adding the VOVGSCSP module, EMA module, and EIou Loss, the model’s performance improves in various aspects, including feature fusion, feature extraction, free variation of aspect ratios in bounding boxes, and addressing the imbalance between easy and hard samples. This performance enhancement is evident from the individual category metrics: in most cases, each added structure leads to improvements in P, R, and AP indicators to some extent. The best indicators are generally achieved by models that have either improved the Neck structure or both the Neck and the loss function, with the latter showing the best overall performance.

In certain categories, there were instances where adding improvement modules resulted in a decline in performance metrics compared to the original model. For example, the P metric for the Short Needle category, as well as the P metric for the Loose Filament Needle category, achieved their optimal values with the original YOLOv8-n. Additionally, the AP metric for the Bent Needle category was best with the YOLOv8n + VOVGSCSP + EMA model. This suggests that the added modules may not be optimally suited for certain categories. For instance, after adding the VOVGSCSP module, the standard convolution operations and depthwise separable convolutions can exchange local feature information across different channels, leading to better detection performance in the Loose Filament Needle category. The EMA module considers information across spatial channels and combines precise spatial structure information with channel information, resulting in notable performance in the Bent Needle category. The use of EIoU Loss allows the model to account for free variation in bounding box aspect ratios and the imbalance between easy and hard samples, leading to improvements in the Squashed Needle, Short Needle, and Normal Needle categories. In summary, it is necessary to analyze the overall effect of the model across the entire dataset.

### Overall effect

**Fig. 12:**
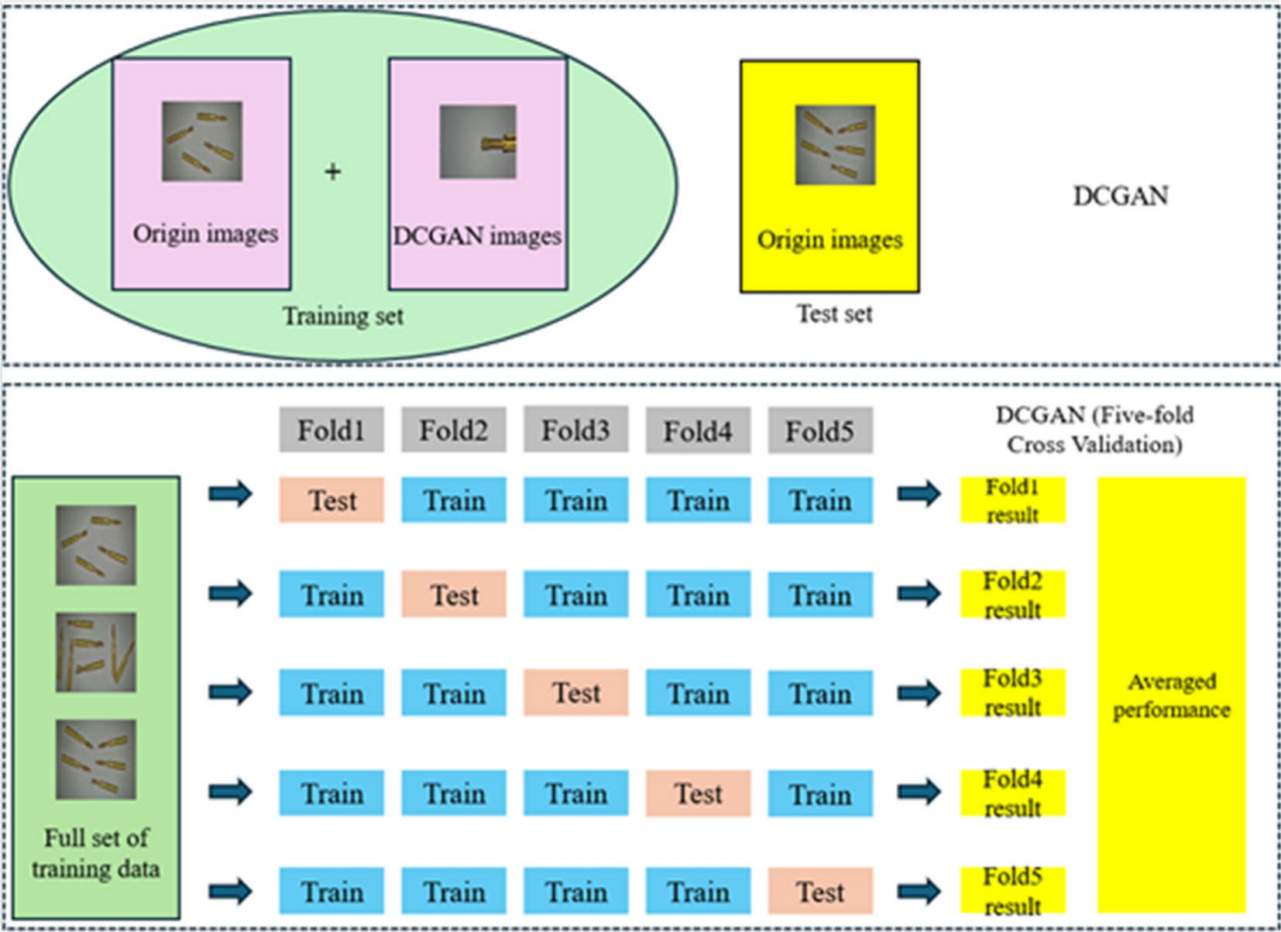
Partitioning method of the experimental dataset.

To validate the feasibility and effectiveness of the improved method, the following approach was taken: The original images were divided into a training set and a testing set. The DCGAN method was used to augment the training samples only within the training set, and the trained model was then tested on the testing set, which did not undergo DCGAN augmentation. Additionally, a 5-fold cross-validation method was employed on the augmented dataset. This approach ensured a more thorough use of the data, with each sample serving as a validation set once. This method helps reduce the randomness from data partitioning and minimizes the risk of overfitting. The dataset partitioning method for the above experiments is illustrated in Fig. 12.

We conducted ablation experiments on three datasets, and the overall performance and six-dimensional metrics of all models are shown in Table 2. Overall, the sequential improvements in the model structure resulted in progressively better performance on the test datasets. In the three dataset partitioning methods, the proposed model showed an increase in the mAP metric by 2.69%, 2.09%, and 3.08%, respectively, compared to the baseline network, while the FPS increased by 36.31%. This indicates that the network significantly improved detection speed and optimized real-time performance, all while achieving superior accuracy compared to the initial model. The proposed model has only 2.668 million parameters and a model size of just 5.486 MB, demonstrating its capability to be deployed on most edge devices with good generalizability. The factors affecting FPS mainly consist of four parts: pre-process, inference, loss, and post-process. The average inference time of the original YOLOv8n on the test set is approximately 4.2ms. Since VEE-YOLO incorporates the VOVGSCSP structural concept, it significantly simplifies the neck part of the detection network and reduces the number of network parameters to some extent, resulting in an average inference time of 2.8ms on the test set. This successfully led to a noticeable improvement in FPS. Additionally, the hardware conditions used in the experiment provided a solid foundation for the detection speed. It is evident that the accuracy of the model using the original dataset, due to the limited number of training samples, is not sufficient for direct industrial application. However, after processing with DCGAN, the model’s detection capability was significantly enhanced. This approach and method of data augmentation, along with the proposed improvements in model feature engineering and loss functions, are feasible and effective.

**Table 2:** Overall indicator improvement effect.

Data set	Indicators	YOLOv8n	YOLOv8n + VOVGSCSP	YOLOv8n + VOVGSCSP + EMA	VEE-YOLO
Origin dataset	P	0.914	0.966	0.924	**0.934**
	R	0.933	0.894	0.913	**0.927**
	mAP	0.817	0.821	0.828	**0.839**
DCGAN	P	0.982	0.983	0.982	**0.985**
	R	0.966	0.982	0.955	**0.986**
	mAP	0.907	0.913	0.921	**0.926**
DCGAN (Five-fold cross validation)	P	0.934	0.969	0.948	**0.961**
	R	0.933	0.946	0.959	**0.974**
	mAP	0.876	0.885	0.891	**0.903**
FPS		179	**334**	244	244
Parameters/million		3.007	**2.667**	2.668	2.668
Model size/MB		6.096	**5.481**	5.486	5.486

Significant values are in bold.

**Fig. 13:**
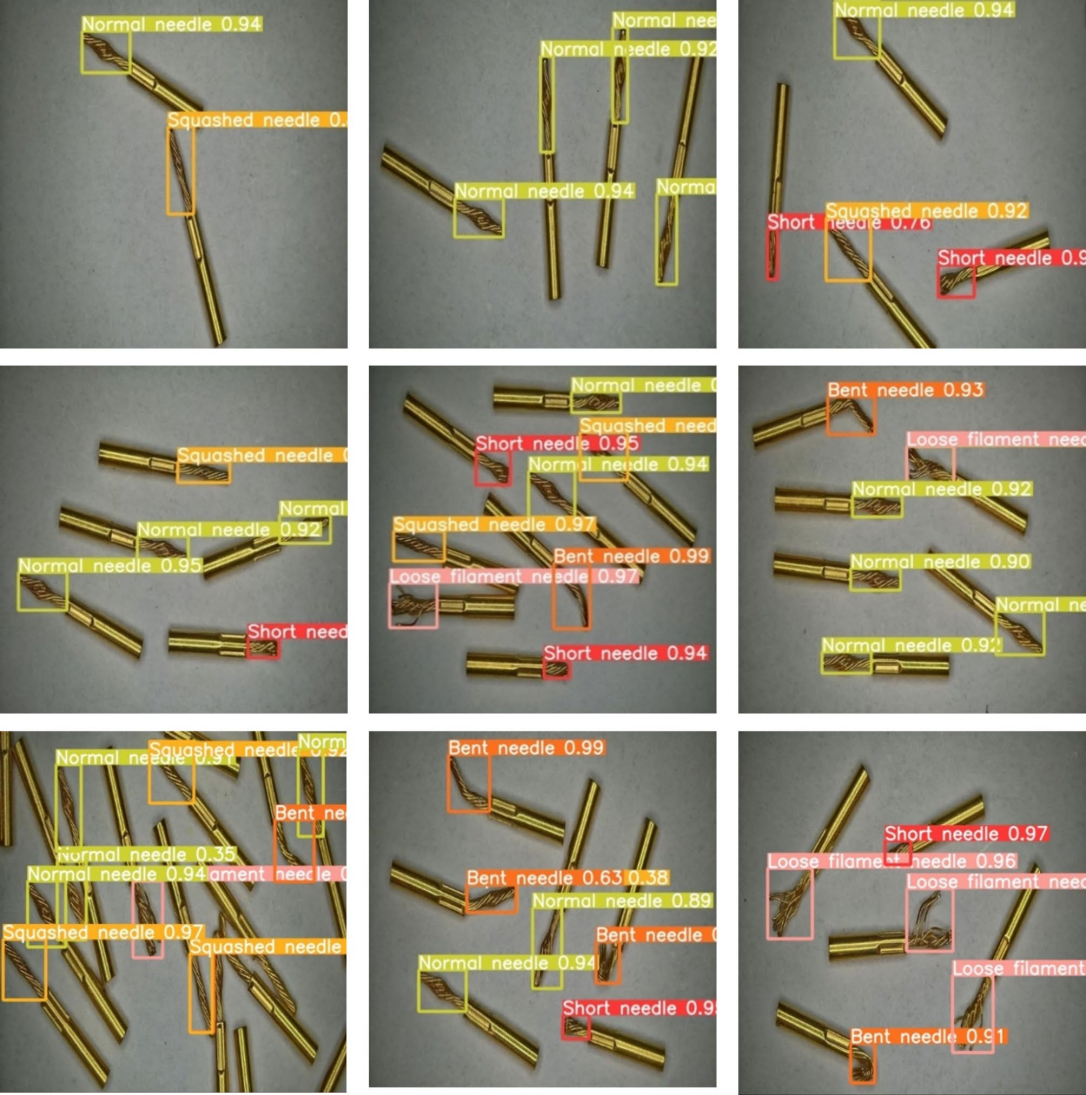
Detection performance illustration.

The selected detection samples in Fig. 13 are all from the test set. From the Figure, the model can accurately detect the corresponding defects in both sparse and dense scenes. The higher confidence scores also indicate that the likelihood of false positives and false negatives occurring has been reduced to a very low probability. However, in cases of high density and heavy occlusion, there may still be instances of false negatives.

### Model performance comparison experiment

**Fig. 14:**
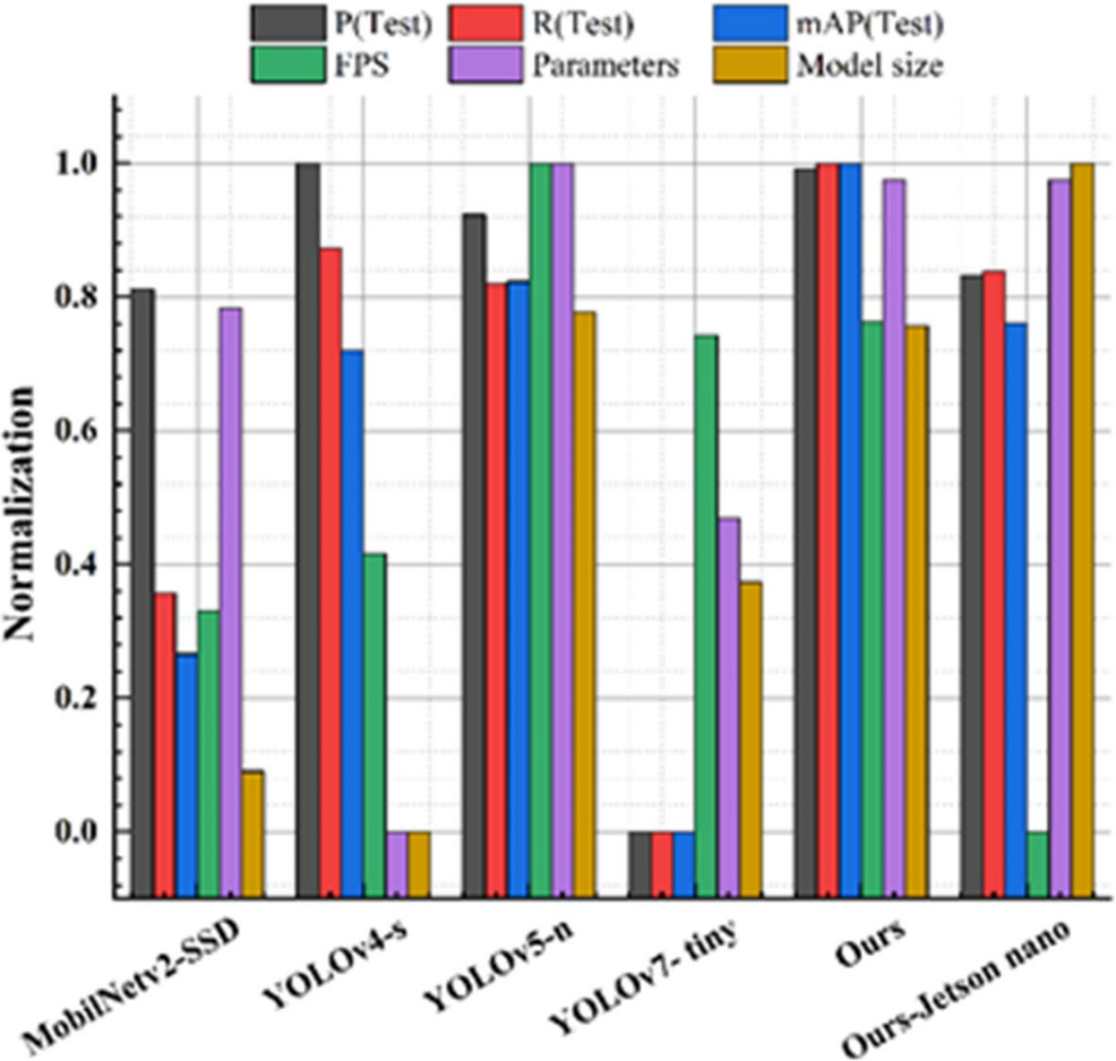
Comparative experimental indicator normalization effect chart.

Deep learning methods in the field of object detection are primarily divided into One-Stage and Two-Stage approaches. Although the latter has an advantage in accuracy, its cumbersome detection process often leads to a long detection time, which is not suitable for real-time detection tasks. Therefore, the One-Stage target detection method that considers both accuracy and real-time performance is widely used in engineering projects. The experiment selected the Yolo series^13–22^ and SSD^40^, which are relatively advanced in the field and have a wide range of applications, as objects to complete the comparative test. Specifically, the study includes models that have been widely applied in various embedded scenarios and have numerous published papers, such as MobileNetv2-SSD^41^, YOLOv4-s^16^, YOLOv5-n^17^, and YOLOv7-tiny^19^. The comparative experiments follow the parameters listed in Table 1 and evaluate the overall performance of the models across six dimensions: P, R, mAP, FPS, parameter count, and model size. Additionally, to validate the model’s deployment effectiveness on the edge, the proposed model is deployed on the Jetson Nano embedded computing platform. TensorRT is used to optimize the model, converting the original .pt format to .engine format, which ensures higher throughput and lower latency during subsequent operations on the embedded development board. All models were trained without the use of pre-trained weights, and they were official versions. The comparative experimental results are shown in Table 3 (Fig. 14).

**Table 3:** Comparison of experimental results.

Data set	Indicators	MobilNetv2-SSD	YOLOv4-s	YOLOv5-*n*	YOLOv7-tiny	Ours	Ours-Jetson nano
Test	P	0.943	**0.987**	0.969	0.754	0.985	0.948
	R	0.815	0.952	0.938	0.720	**0.986**	0.943
	mAP	0.727	0.850	0.878	0.655	**0.926**	0.861
FPS		118	143	**313**	238	244	22
Parameters/million		3.94	9.121	**2.504**	6.017	2.668	2.668
Model size/MB		16.723	18.256	**5.142**	11.947	5.486	1.372

Significant values are in bold.

First, YOLOv7-tiny and MobilNetv2-SSD both exhibited poor performance on this dataset. The worst-performing model, YOLOv7-tiny, achieved a mAP of only 0.655, while the top-performing improved model showed significant improvements in mAP compared to YOLOv7-tiny and MobilNetv2-SSD, with increases of 41.37% and 27.37%, respectively. This demonstrates the substantial enhancement in detection accuracy achieved by the improved models over the YOLOv7-tiny and MobilNetv2-SSD models. Both YOLOv7-tiny and MobilNetv2-SSD models exhibited lower rankings in terms of FPS, parameters, and model size, and only had an advantage over YOLOv4-tiny, with MobilNetv2-SSD having the poorest FPS performance among the models tested. This suggests that the improved models strike a better balance between accuracy and computational efficiency, making them more suitable for real-time detection tasks compared to YOLOv7-tiny and MobilNetv2-SSD. In Table 3, the P index for YOLOv7-tiny is only 0.754, indicating that this model struggles to correctly classify a significant number of samples. This situation suggests that YOLOv7-tiny and MobilNetv2-SSD have limited feature learning capabilities when it comes to the defect samples of stranded elastic needles. These models might not be suitable for defect detection tasks in complex scenarios.

Second, YOLOv4-tiny shows a mediocre overall performance. Despite having the highest number of parameters and being the largest model in the experiment, YOLOv4-tiny performs better than MobilNetv2-SSD and YOLOv7-tiny in the detection task but still falls short of the improved models. Although YOLOv4-tiny shows acceptable detection accuracy, YOLOv5-n and the improved model proposed in this paper exhibit stronger overall performance and are therefore more suitable for such applications compared to YOLOv4-tiny.

Third, YOLOv5-n has achieved relatively excellent performance. After several official updates, its performance has improved significantly. YOLOv5-n exhibits a balanced state between P and R, with detection rates and accuracies only slightly lower than VEE-YOLO. Especially in terms of FPS, Parameters, and Model size, YOLOv5-n outperforms the compared models, except for a slightly lower FPS than VEE-YOLO. Therefore, YOLOv5-n is also suitable for defect detection tasks in engineering scenarios.

Fourth, VEE-YOLO performs impressively. Compared to numerous models, both the R and mAP metrics are superior. Additionally, the model exhibits excellent FPS performance (244). This indicates that the model has achieved the best results in both detection accuracy and speed. Parameters and model size are also in the top tier, demonstrating the model’s superior overall performance. This model accurately recognizes defect types, performs detection rapidly, and demonstrates robustness and practicality. Furthermore, the model deployed on the Jetson Nano uses TensorRT acceleration to meet the real-time requirements of edge devices. While there is a slight reduction in accuracy, the model’s size is also reduced. This ensures that the model’s accuracy remains superior to that of MobileNetV2-SSD, YOLOv4-s, and YOLOv7-tiny, with an FPS still reaching 22, making it suitable for practical engineering deployment.

**Fig. 15:**
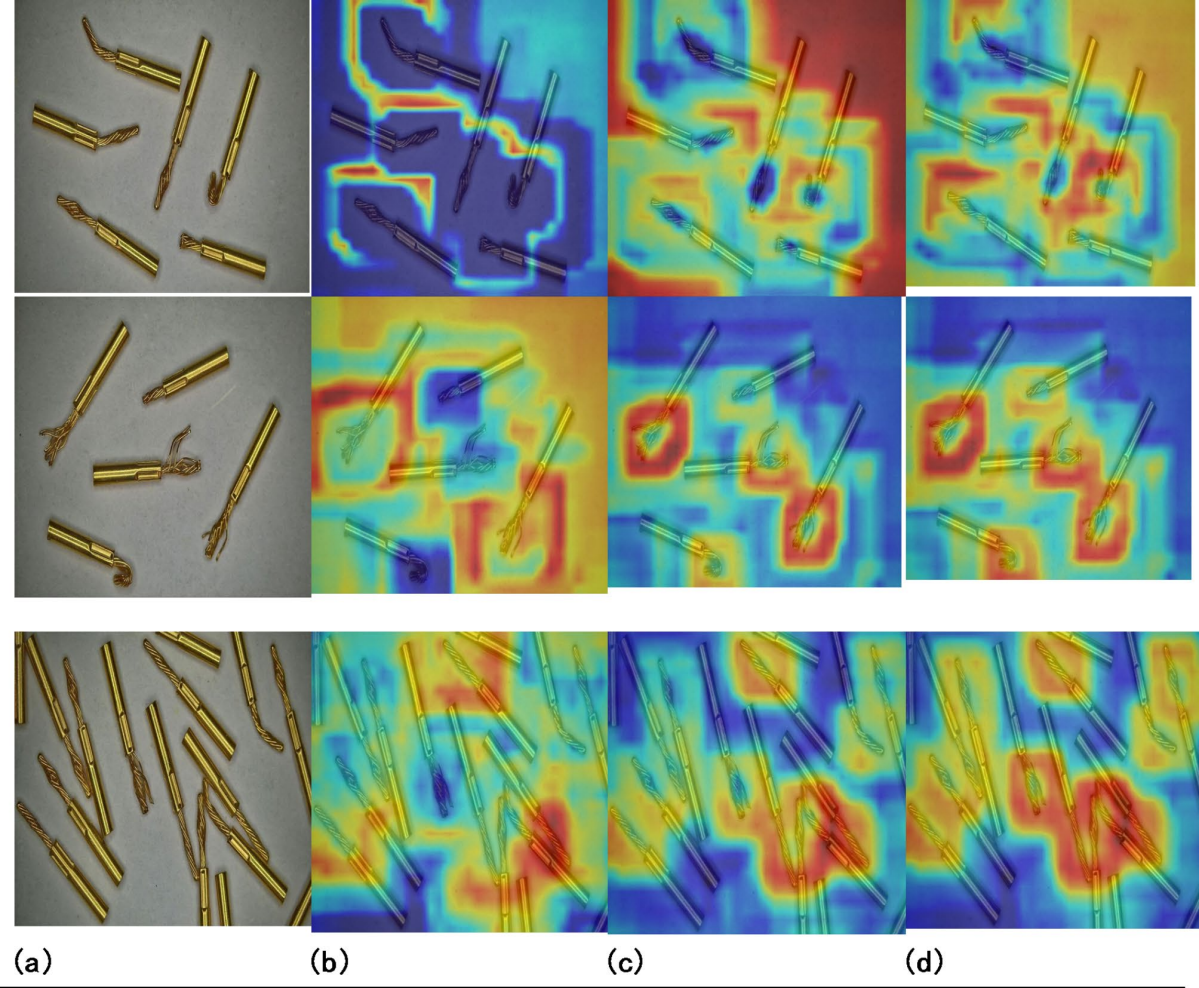
Grad-CAM visualization. (**a**) Original image. (**b**) YOLOv4-tiny Grad-CAM map. (**c**) YOLOv7-tiny Grad-CAM map. (**d**) VEE-YOLO Grad-CAM map.

To enhance the interpretability of the model’s performance, Grad-CAM^42^ map of YOLOv4-tiny, YOLOv7-tiny, and VEE-YOLO are compared on the same original images, as depicted in Fig. 15. Grad-CAM is based on the gradient calculated by backpropagation of class confidence scores and generates corresponding weights. Since the weight contains category information, it has great positive significance for the final detection performance. the Grad-CAM map of the model in this paper is the best, and the dark red parts (focus areas) are the same type of targets. That aligns with the initial intention of designing a model to detect small objects. In summary, the model presented in this paper has achieved the best results while maintaining interpretability.

## Discussion

This study proposes the VEE-YOLO model to address defect detection in precision components under complex industrial scenarios. The model incorporates improvements to existing methods in terms of data augmentation, feature extraction, feature fusion, and loss functions. A dataset of stranded elastic needle defects was specifically created based on real-world conditions to validate the proposed model. Experimental results demonstrate that the VEE-YOLO model achieves significant performance on small-sample defect datasets, attaining a detection accuracy of 0.926 and an FPS of 244. Compared to the baseline model, the VEE-YOLO model improves both detection accuracy and speed, with the mAP increasing by 1.32% and FPS improving by 86.59%. The network achieves optimal accuracy while significantly enhancing detection speed and real-time performance. Among four comparative algorithms, the proposed model achieves the best results in Recall (R) and mAP metrics, showcasing its accurate defect classification, rapid detection process, robust performance, and practical applicability.

The improvement in P, R, mAP, and FPS metrics achieved by this work can be attributed to the following contributions: First, the dataset of stranded elastic needle defects was augmented using a Generative Adversarial Network, ensuring inter-class fairness, which significantly enhanced the model’s detection capability. Second, by incorporating Depthwise Separable Convolutions (DSC) and utilizing GSConv as the basic unit, the GS bottleneck was designed. Coupled with the VOVGSCSP structure for feature fusion, this approach reduced network parameters while strengthening feature engineering, thereby improving detection accuracy. Third, cross-dimensional interactions contributed to channel and spatial attention prediction. The integration of EMA enriched the network’s feature extraction, combining precise spatial structural information with channel information to enhance the quality of feature engineering for defect detection. Lastly, the adoption of EIoU Loss addressed issues related to the free variation of bounding box aspect ratios and the imbalance of easy and hard samples, improving the overall performance of the detection task. The results in Tables 2 and 3, along with Figs. 10, 11 and 14, and 15, validate these advancements.

Many studies have reported improvements to the Backbone, Neck, and Head of advanced models like YOLOv8n, yielding varying degrees of enhancement in object detection accuracy. For example, in reference^43^, YOLOv8-Adamas was developed based on the YOLOv8n detection algorithm for synthetic diamond quality assessment. Using the ConvNeXt V2 architecture to redesign the Backbone and incorporating the Dyhead-Detect dynamic detection head, detection accuracy was improved to 0.947. Reference^44^ introduced AGW-YOLOv8 for underwater object detection, enhancing the Backbone with the convolutional block attention module (CBAM) and integrating GSConv modules and Squeeze-and-Excitation (SE) attention mechanisms into the Neck, achieving a detection accuracy of 0.829. Reference^45^ developed YOLOv8-ADSC for helmet detection in complex operational environments. The content-aware reassembly of features (CARAFE) module replaced the Upsample module, while adaptive spatial feature fusion (ASFF) and deformable convolutional network version 2 (DCNv2) were integrated into the detection head, achieving a detection accuracy of 0.342. Our research further confirms that reasonable modification strategies can effectively enhance network model detection accuracy. However, the aforementioned studies often introduced more complex network architectures, increasing computational resource consumption and reducing detection speed compared to the YOLOv8n baseline. Our study shows that incorporating DSC and VOVGSCSP into the Neck reduces model parameters while achieving high-quality feature extraction, improving detection speed. By combining the focusing capability of attention mechanisms and the optimization guidance of the loss function, the model enhances its ability to capture critical data information while reducing unnecessary computational resource usage, resulting in improvements in both detection accuracy and speed.

Despite the promising results of the proposed method in small-sample twisted needle defect detection, it still relies on labeled data for network training. Additionally, the use of adversarial data augmentation methods may face challenges such as training instability and high computational costs. These issues remain significant challenges for zero-shot or few-shot defect detection tasks in industrial domains.

## Conclusion and future works

The VEE-YOLO model we propose effectively addresses the defect detection of precision parts in complex industrial scenarios. It can be deployed on edge devices without compromising detection accuracy, providing new insights for defect detection methods in the industrial field. For future complex defect detection tasks, in-depth research can be conducted in the following areas:

1. Large-scale data annotation is expensive and time-consuming, and acquiring large amounts of annotated data in the industrial field is difficult, if not unrealistic. Future research can focus on unsupervised and semi-supervised learning methods, enabling models to learn effectively without extensive labeled data.
2. Transfer learning allows defect detection models to be transferred from one domain to another, especially in cases where defect types are variable or data is scarce. Future work can explore the adaptability of transfer learning techniques across different industrial applications.
3. Complex defects often exhibit different characteristics at varying scales and angles. Multi-scale and multi-angle image processing techniques can help more accurately identify hidden defects. Future research can focus on extracting useful features from multidimensional data to enhance detection accuracy.

## Data Availability

The datasets used during the current study are available from the corresponding author upon request.
